# Reversible and tunable photochemical switch based on plasmonic structure

**DOI:** 10.1038/s41598-020-62058-z

**Published:** 2020-03-20

**Authors:** H. Mbarak, R. Taheri Ghahrizjani, S. M. Hamidi, E. Mohajerani, Y. Zaatar

**Affiliations:** 1 Laser and plasma Research Institute, Shahid Beheshti University, G. C. Tehran, Iran; 2 Faculty of Sciences 2, LPA, Lebanese University, BP 90656 Jdeidet, Lebanon

**Keywords:** Materials for optics, Nanoscale devices

## Abstract

For the first time, pyranine (8-hydroxypyrene-1,3,6-trisulfonate, HPTS) is studied for realizing active plasmonic control, which is attracted considerable attention owing to its unique photophysical and photochemical properties. We have used this photoacid (HPTS) as an active surrounding medium that can be optically controlled and used for modulating plasmon resonances. In this paper, the fabrication of 2D-plasmonic grating coated by thin film of HPTS exposed to UV irradiation is reported. By switching the UV light on and off, the HPTS thin film maintains an excited-state proton transfer (ESPT) process followed by green fluorescence resulting in a plasmonic redshift caused by the variation of the refractive index. Furthermore, this photochemical active medium has also played another important role in plasmonic sensing, in which the emission-based response of HPTS thin film in 2D-plasmonic grating to water vapor upon photoexcitation is demonstrated, for both s and p polarizations. This tunable, flexible and reversible light-driven system will enhance the development of active plasmonic structures and will have a great influence on many fields such as, biochemical optical sensors and all-optical plasmonic circuits.

## Introduction

Active plasmonics is a promising field of science and technology due to the capability of exploiting the active control of surface plasmon resonance (SPR)^1^. To date, the prospering development of active plasmonic structures has been attracted a swiftly rising research for solving many different problems encountered by our world, in which their control can be achieved by different driving methods such as light^2^, electric field^3^, temperature^4,5^, etc.

Furthermore, based on the sensitive dependence of the dielectric function of the surrounding medium on the SPR, tuning the refractive index can be considered as an essential way to achieve the active control of SPRs^6^. For this reason, choosing a suitable plasmonic structure by essential response in the required spectral region as well as an active medium with tunable dielectric responses under external fields^7^ in new plasmonic structures, was and is still the focus of researchers to find a novelty for developing the active plasmonic devices.

Actually, among all external fields used for plasmonic structures in tunable dielectric surroundings, all-optical control is much desired in this domain, especially UV light owing to many advantages including the non-contact tuning, low power consumptions, fast switching speed and the facility of integrating in future all-optical plasmonic circuits^8,9^.

Until now, many studies on all-optical plasmonic switches have been addressed. Among these, light-driven plasmonic switch based on Au nano disk arrays embedded in photo-responsive liquid crystals^10^. Another one based on Azo-dye-doped holographic polymer-disperse liquid crystals (HPDLC) in a hybrid system composed of photoswitchable gratings and Au nanodisk arrays^11^.

In this work, we have studied a reversible and tunable photochemical switch based on 2D plasmonic grating structure, which enabled us to realize the modulation of surface lattice resonance of plasmonic periodic arrays in the vicinity of photo sensitive dyes.

Based on photo induced charge transfer effect in these photosensitive dyes, one can find new mechanism to control optical activity, switching, nonlinear properties like as two photon absorption and so on^12,13^.

Between several photochromic molecules, HPTS as a new candidate is examined for the first time as a tunable active medium under UV irradiation for controlling the plasmonic properties of the 2D-plasmonic grating system. HPTS has been used before to study the excited-state proton transfer (ESPT) process, in which upon photoexcitation the photoabsorption can take place near 400 nm^14^ inducing a vertical transition between the ground state and the electronic excited state followed by fluorescence and electronic absorption changes. By this manner, the refractive index changes creating an intensity variation in addition to a plasmonic shift in the reflectance spectrum can realize the tunability of the surface plasmon resonance and all optical switches. Moreover, the molecule of HPTS has an OH-group on one of their four aromatic rings making it favorable for different sensing applications such as carbone dioxide^15^, biosensing^16^, pH sensing^17^ and humidity^18^.

In the other hand, our fabricated plasmonic structure is characterized by its simple fabrication and reasonable price in which it has drawn a good attraction by other groups in different domains in plasmonics such as random lasing, biomedical substrate and also temperature confinement^19,20^.

Now, by combining the plasmonic properties of the 2D gold nanograting and the optical properties of the fluorescent dye, the plasmonic system device based-HPTS will serve as a new all-optical plasmonic device that can be applicable in important applications in switching and sensing.

## Experimental part

Fabrication of 2D plasmonic grating is successfully completed using nanoimprint lithography method. Details of this method and the fabrication process to produce plasmonic structure are described in our previous work^3^.After deposition of the gold layer and attaining two dimensional (2D) plasmonic structure, a thin film of 8-Hydroxypyrene-1,3,6-trisulfonic (HPTS) is coated on, which allows the definition of switching under UV radiation that supposed leading to a good tuning of the plasmonic properties of the structure.

**Figure 1:**
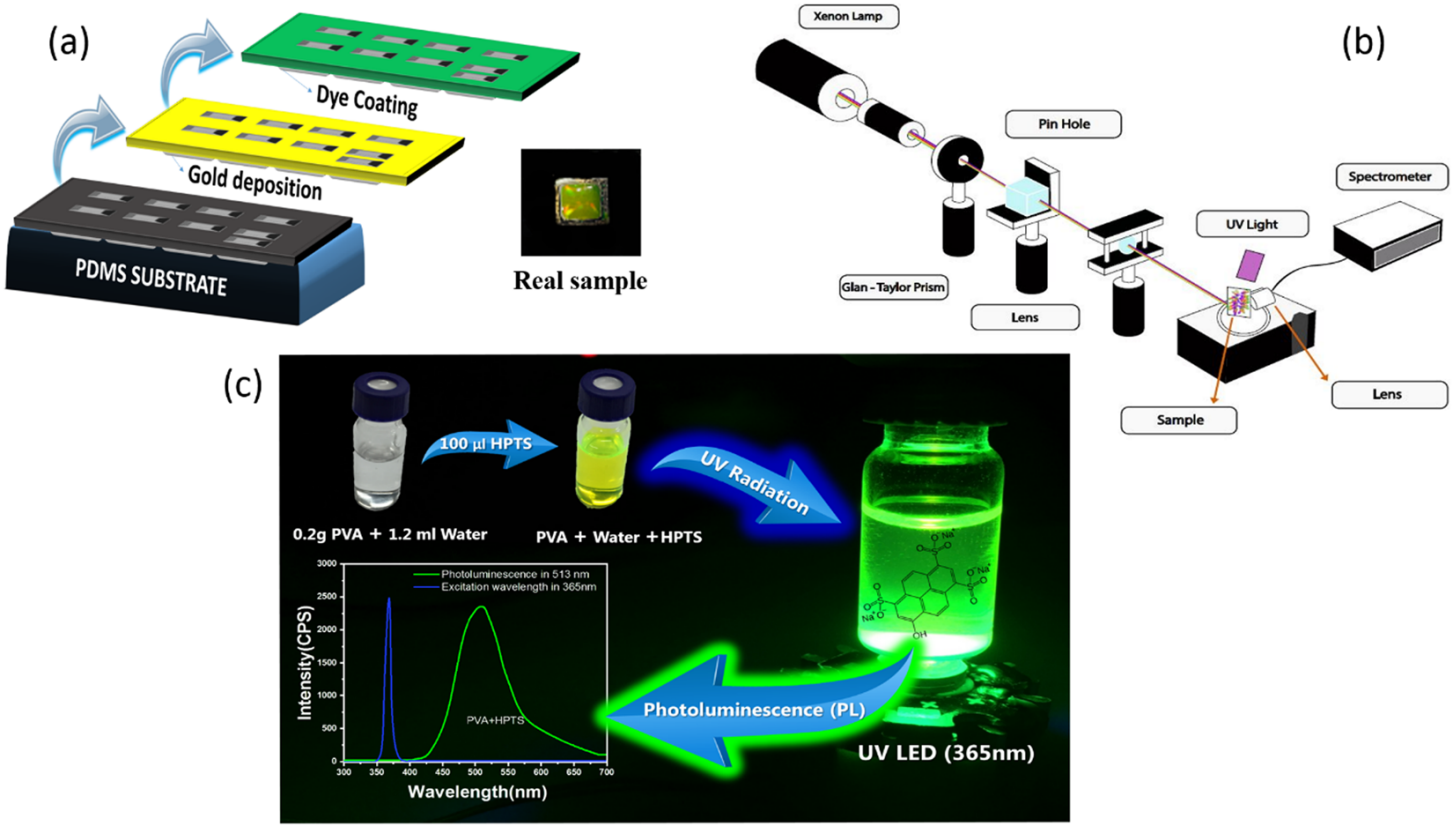
(**a**) Schematic diagram of sample preparation and real image of the prepared sample, (**b**) experimental setup to record the switching manner and (**c**) procedure of mixture preparation and the fluorescence of prepared mixture as over layer.

The procedure of the fabrication of the mixture, multilayer and also the real image of the prepared sample is schematically described in Fig. 1(a,b) respectively. We are used the compound HPTS as our principal active medium which act as a fluorescent water-soluble pyrene dye. Thus, a 0.2 g of polyvinyl alcohol (PVA) was dissolved in 1.5 ml deionized water on a heater at temperature of 60 °C. Then, a 100 µL of HPTS is added to the mixture.

As a harmonious guest, PVA was chosen to be present in the solution, due to its strong interaction with both HPTS and water simultaneously, which makes the mixture more viscous and therefore an easiest coating of the dye on the gold surface can be obtained.

To get the switching manner under UV irradiation, we use experimental setup as shown in Fig. 1(c). This figure shows the experimental setup for optical reflection spectra measurements, which are recorded for different polarizations of light (S and P polarizations) at a specific angle of the sample under UV irradiation.

The source of radiation is a Xenon Lamp operating in the range of 400 to 800 nm. The polarized light (S or P) focused on the surface of the sample is reflected at an incidence angle of 54 degrees which satisfied the surface lattice resonance for the sample and then recorded with a spectrometer (HR4000G-UV-NIR from Ocean Optics), after passing through a high magnification objective lens coupled with an optical fiber.

In addition, as a pump light, we use 365 nm ultraviolet LED at normal incidence in front of the sample.

In fact, the photo-responsive HPTS used in this solution is selected to be our active medium of this work owing to its unique photophysical and photochemical properties. Such pyranine HPTS exhibits a strong absorption near 400 nm and can be excited using UV light as it schematically shown in Fig. 1(b)^14^. Finally, the photoswitching process were considered in the exposure of the sample by humidity.

## Results and discussion

**Figure 2:**
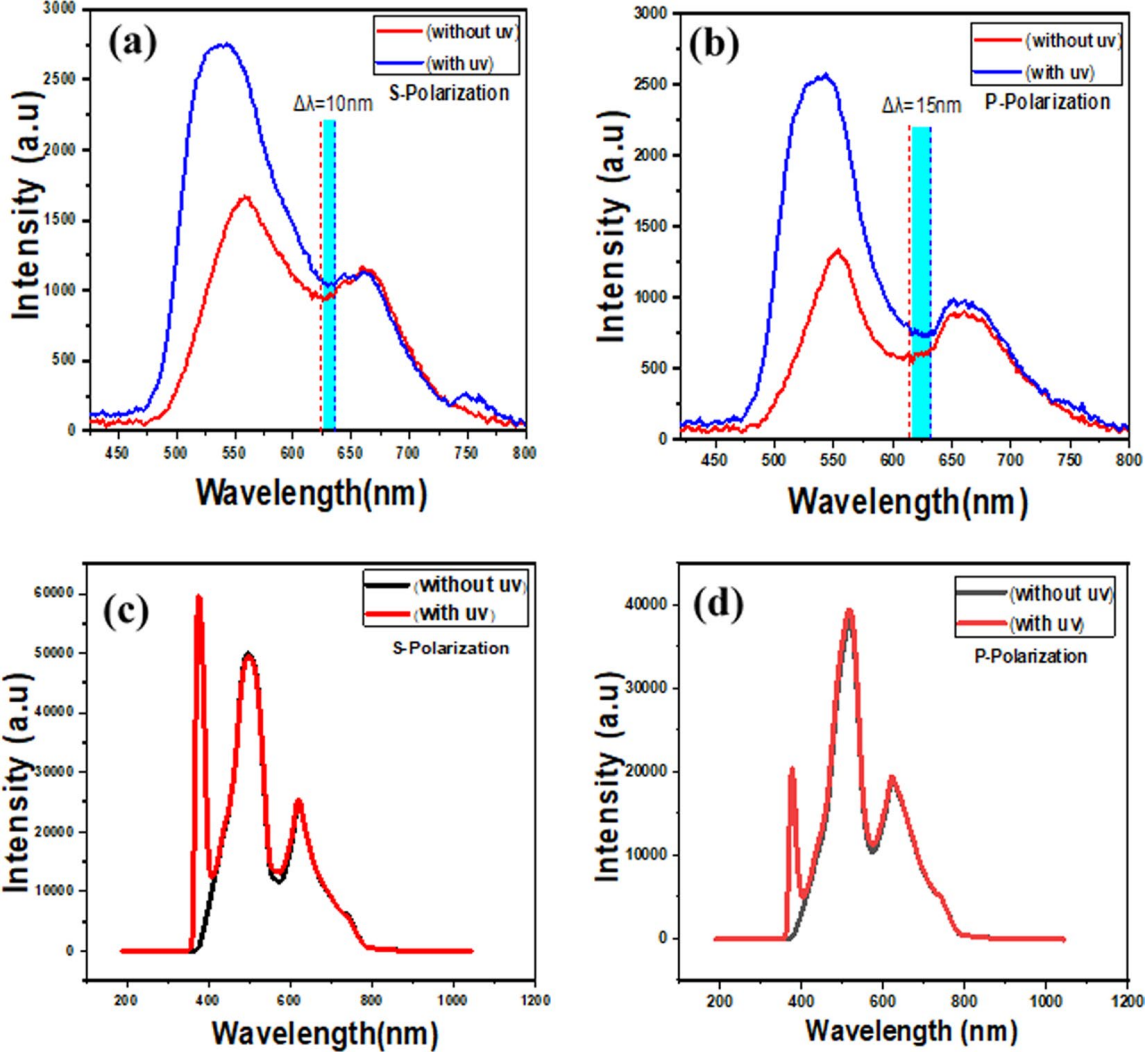
Reflectance spectra of the sample (**a** and **b**) plasmonic sample with HPTS cover layer (**c** and **d**) plasmonic sample without HPTS cover layer, for both polarizations.

Reflection spectra under the probe light and at a fixed incidence angle (54°), before and after active medium’s coating (HPTS) on the gold surface of the sample with and without UV radiation, are recorded for both s and p polarizations, as shown in Fig. 2(a, d).

It should be noted that our plasmonic structure is formed by gold nano arrays that are arranged in a wavelength scale array, in which the array period is comparable to the wavelength of the particle plasmon resonance and therefore two important hybridized modes can exist: The narrow diffracted orders (DOs) and Localized surface plasmons resonance (LSPRs) in far-field measurements^21^. Furthermore, when the LSPRs associating with individual nano rods couple the diffracted waves propagating in planar surface, an exciting phenomenon can take place, also referred to as surface lattice resonance (SLRs)^3,20^.

Since the surface lattice resonance is a result of the coupling between LSPRs and DOs, the spectral position of the resonance wavelength of the SLR-based 2D grating is strongly sensitive to the refractive index of the surrounding medium, because according to Mie theory, if the refractive index of the local medium is varied, the LSPR should be affected.

Therefore, this relationship can serve as an important basis to understand fundamental principles of SLRs active control which it can be exhibited by modulation depth and shift of the reflected light from the sample.

**Figure 3:**
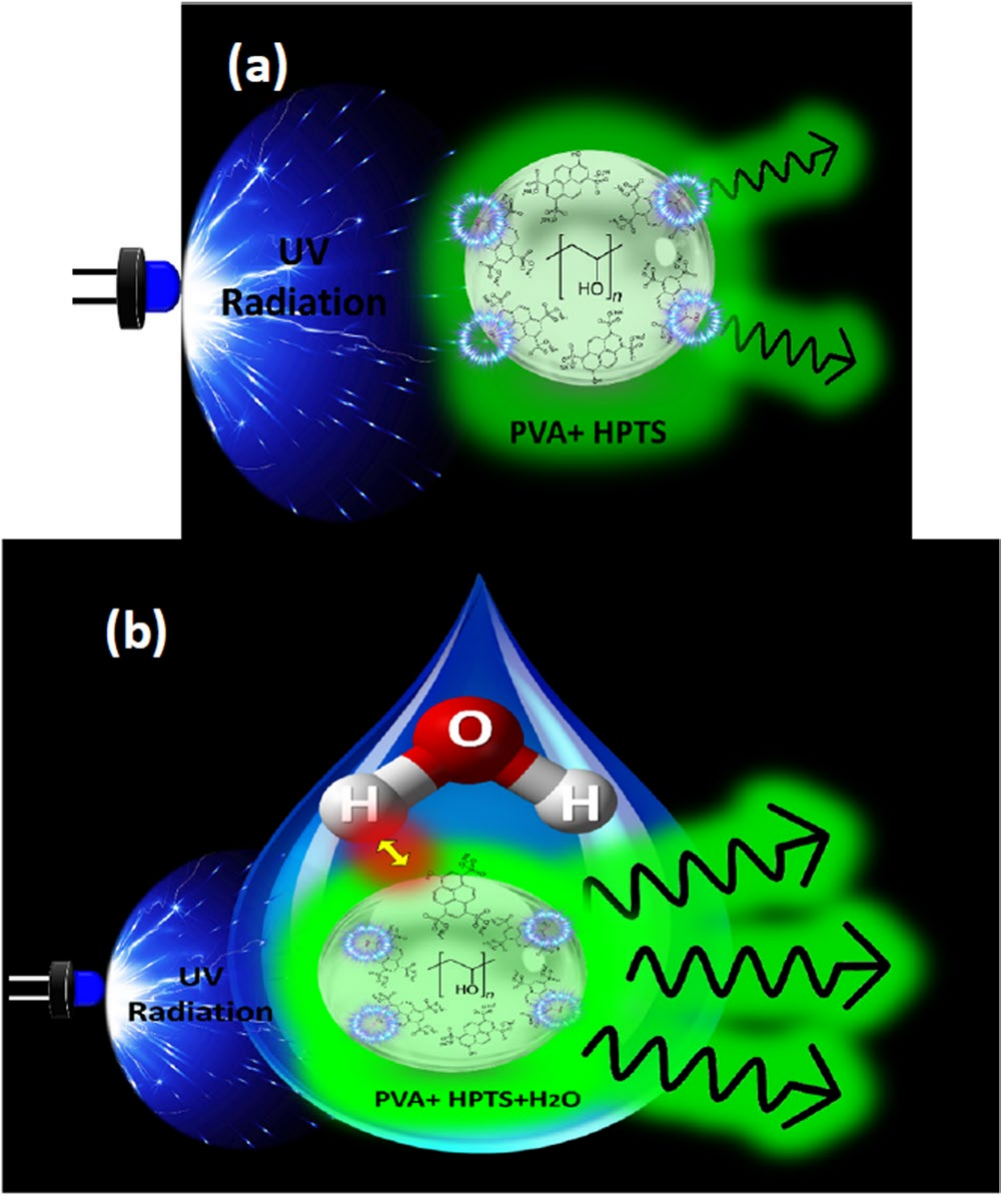
Schematic picture represents the ESPT process of HPTS upon UV irradiation followed by fluorescence (**a**) without H_2_O humidity (**b**) with H_2_Ohumidity.

Upon photoexcitation of HPTS by UV led, absorption of a photon can hugely increase the acidity of a molecule and an intermolecular excited-state proton transfer (ESPT) process can be triggered, in which the HPTS molecules in the excited state dissociate by donating a proton to surrounding water molecules and converting to the photobase form, followed by green fluorescence peaked at^21–23^. The schematic picture of proton transfers from HPTS to water solvent under UV irradiation resulting in migration of protonic charge and radiative green emission in the absence and presence of H_2_O humidity is shown in Fig. 3(a,b), respectively.

Consequently, the electronic absorption changes and the fluorescence, generated from the EPST process upon photoexcitation, directly lead to a change in the refractive index of the local medium (HPTS thin film) coated on the gold surface of the plasmonic structure and therefore a significant modulation of the optical plasmonic properties of the system can be achieved owing to the essential dependence of the LSPR on the refractive index of the surrounding medium.

It is not surprising to use water as a best solvent for HPTS due to many factors, including EPST process only occurs in water^24^ and therefore the fluorescence takes place at approximately 513 nm, in which it can be considered very nearly to the surface lattice resonance wavelength. In addition, owing to the three sulfonic acid groups that contains HPTS, an excellent solubility of HPTS molecules can be obtained^14^.

Furthermore, when the gold surface is coated by HPTS, the reflection spectra, as we mentioned above, are established under UV irradiation for both s and p polarizations, as shown in Fig. 2(a,b) respectively. It can be noticed from this figure that a redshift associated with an increasing of the intensity is obtained (for s polarization and for p polarization). Actually, to confirm that the obtained shift is coming from the effect of UV irradiation on the polymer, we are recorded the reflection spectra of an original sample without pyranine exposed under UV light, see Fig. 2[c,d]. We can see that there is no shift of the spectral position of the resonance wavelength when the UV light is turned ON, which gives that the polymer itself is responsible about this shift, and by this way the plasmonic proprieties of the structure can be tuned.

**Figure 4:**
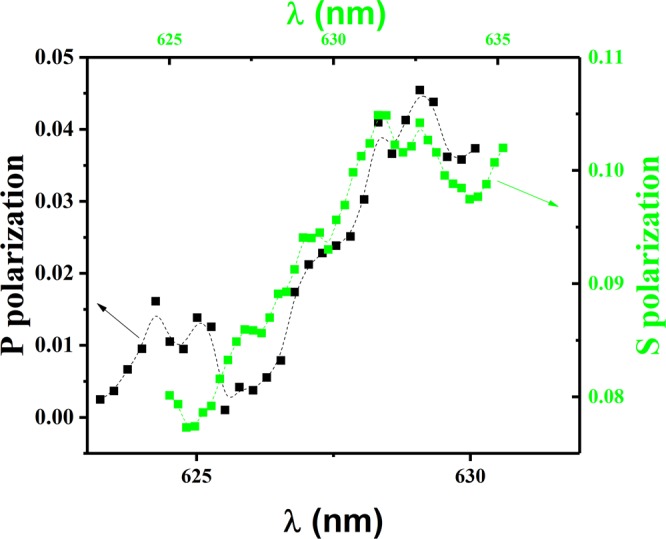
Difference in the real part of the refractive index with and without UV radiation for both p and s polarizations.

On the other hand, the obtained redshift itself can be elucidated by the increasing of the refractive index of the surrounding medium (thin film of HPTS) under UV radiation, which was replaced by air on the gold surface of the plasmonic structure. This fact was confirmed by the aid of derivation the refractive indices of the samples with and without UV radiation by Kramers-Kronig relation^25^ as shown in Fig. 4 for both polarizations, which indicates enhancement in the vicinity of red shift of the SLR in each polarization as can be seen in selected area of Fig. 2(a,b).

**Figure 5:**
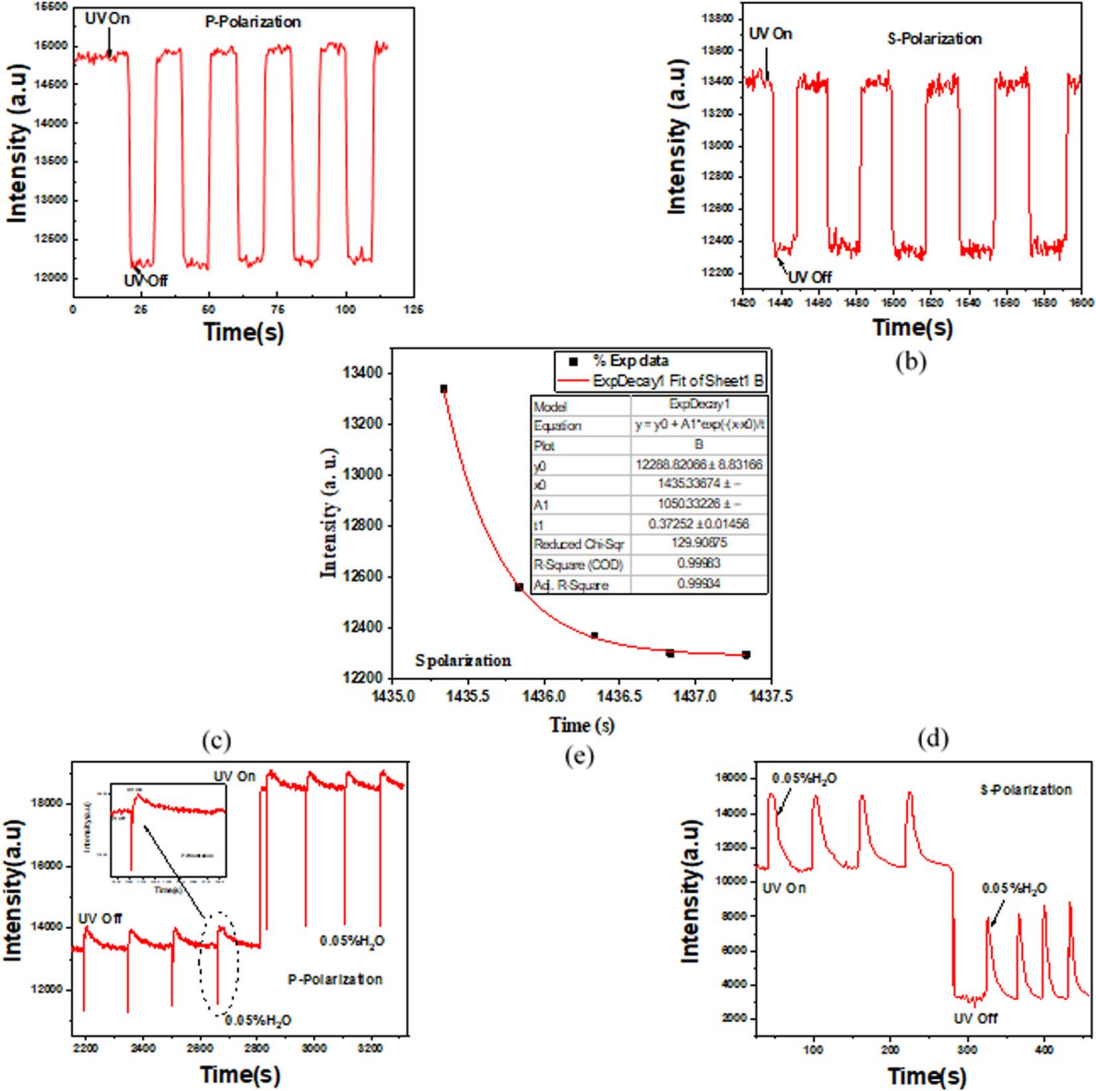
Emission-based response of HPTS thin film in 2D-plasmoinc grating (**a**) and (**b**) upon photoexcitation by UV light and (**c**) and (**d**) to H_2_O humidity for s and p polarization.; (**e**) the response time for S polarization.

Now after approving the effect of UV light onto the SLR and also the refractive indices, we want to characterize the switching effect of the system under UV Led (365 nm) at normal incidence. UV light was mutually switched ON and OFF, for both s and p polarizations light, leading to a variation in the reflection intensity as shown in Fig. 5 (a,b) for both polarizations. Actually, in the ON state of UV light, the reflectance intensity is increased, whereas when the LED is turned off, a decreasing of the intensity can be observed for both polarizations. It indicates that as we mentioned above, by irradiating the molecules of HPTS by UV light, they excite from the ground state to an excited electronic state, exhibiting excited-state proton transfer (HPTS) accompanied by a radiative emission and therefore leading to an enhancement in the reflection intensity versus time.

This fluorescence signal change caused by these solvation events upon the photoswitching is also correlative to the redshift observed above, providing a clear guideline for tuning the switchable plasmonic properties of the system.

It can be noted, that the repetition of the reversible switching process was realized many times and since the intensity variation looks like the same through many periods, the reversibility and the reliability of the refractive index change of the mixture can takes place. The response time of the photoswitching process is considered to be in the order of 10^2^ ms for s polarization, as shown in Fig. 5(e), which is fitted with a first order exponential decay according to the expression below

1 $$y={y}_{0}+\,{A}_{1}{e}^{\frac{-(x-{x}_{0})}{{t}_{1}}}$$

where y_0_ and *x*_0_ are the width of origin sets to ∼12289 and ∼1435 in y and x axis respectively. *A*_1_ is the amplitude of the decay function set to ∼1050 and *t*_1_ is the response time of the system which predestined at ∼372 ms.

In addition to the function of plasmonic switchable modulation, HPTS plays a key role in plasmonic sensing, especially in water vapor. Subsequently, as a first attempt, the sensing functionality of 2D-plasmonic grating using tuning active medium (HPTS) was successfully performed. The emission-based response of thin film sensing of HPTS, coated on the gold surface of the plasmonic structure, to water vapor (0.05% H_2_O) with and without UV irradiation is shown in Fig. 5(c,d) for p and s polarizations respectively.

The underlying sensing mechanism could be described by this way: First, the fluorescence signal change (photoswitching between ON and OFF states of UV) of the reflectance intensity versus time for the sample without water vapor is demonstrated and described above. Now, during the exposure of the sample to water vapor, a significant increasing of the fluorescent signal is observed, regardless on the presence or absence of the UV radiation. But, when the water vapor stream is disconnected, the fluorescence is reduced and experiments showed that the recovery time was longer when the sensor was exposed to UV than there was no ultraviolet radiation at the surface sensor. Obviously, upon photoexcitation of UV, automatically the ESPT process should take place resulting in the change of the form of HPTS from protonated state to deprotonated state and therefore during the exposure of water vapor, the sample must be more capable of absorbing water vapor molecules due to the hydrogen bond weakening presented in the O–H group, which can accelerate the process and change the fluorescence. For this reason, the long recovery time can be assigned by the reversible behavior taken to return back to its original state when the water vapor is disconnected.

Furthermore, in spite of this variable manner of exposed sample under H_2_O, under UV and without UV irradiation, there is another fantastic difference between both polarizations. As it can be seen from Fig. 5 (d,e), we have schottky barrier like response for p polarizations when the sample wants to come back at rest after H_2_O exposure turns off. This is common manner due to the neighboring of gold nanostructure and HPTS cover layer as metal and semiconductor media.

## Conclusion

In summary, in accordance with the previous work, we have been able to realize an all-optical plasmonic switch based on photochemical material (HPTS), which has the ability to control the plasmonic properties of the system upon photoirradiation (UV Led). Switching the UV light ON the molecules of HPTS, after absorbing a photon, excite from a ground state to an excite state resulting in a proton transfer to water solvent, followed by a green fluorescence which induces a change in the refractive index and thus affecting the tuning of the optical plasmonic properties of the structure. HPTS is directly coated on a gold surface deposited on PDMS substrate, which is simply fabricated using Nanoimprint lithography method. Our results show efficient photoswitching process by 270 ms for s polarization and also good sensitivity to the H_2_O exposure in both polarizations. This flexible system is attracted by others for many fields especially in biosensing and randon lasing, and now we have successfully demonstrated its efficiency in water vapor sensing at a first step, that it can be developed in future work.
